# Instability in the environment and children’s in-school self-regulatory behaviors

**DOI:** 10.3389/fpsyg.2025.1498961

**Published:** 2025-03-18

**Authors:** Karen E. Smith, Stephanie J. Dimitroff, Kelly E. Faig, Emily M. Silver, Greg J. Norman

**Affiliations:** Division of Social Sciences, Department of Psychology, University of Chicago, Chicago, IL, United States

**Keywords:** early life stress, self-regulation, environmental stability, longitudinal, children, adversity

## Abstract

**Introduction:**

Experiences of chronic and/or extreme stress early in childhood are associated with altered self-regulatory behaviors. However, there is a range of variability in children’s behavioral outcomes after experiences of stress. Understanding what contributes to this variability in children’s responses to stress can aid in the development of more effective programs aimed at supporting children’s self-regulatory processes. The current study examined relationships between indices of environmental stability and changes in children’s self-regulatory behaviors.

**Methods:**

Ratings of children’s self-regulatory behavior were collected in collaboration with a school program once a month over the course of the academic year. Measures of environmental stability were collected for each child.

**Results:**

Children demonstrated increases in self-regulatory behaviors over the course of the study. Additionally, children in home environments characterized by high levels of environmental instability demonstrated greater positive behavior change during the program.

**Discussion:**

This study suggests that there are important individual differences in children’s patterns of self-regulatory behavior changes, and points to complex interactions between children’s home environment, implementation of a more positive and stable environment, and changes in behavior.

## Introduction

Chronic or extreme stress in childhood is associated with a wide range of long-term effects on development, including increased risk for negative physical and mental health outcomes and alterations in learning, emotion processes, and behavior (Pechtel and Pizzagalli, 2010; Lupien et al., 2009). One prevalent outcome associated with stress in childhood is disruptions in children’s self-regulatory behaviors (Palacios-Barrios and Hanson, 2019). However, not all children who experience chronic or extreme stress demonstrate problems with self-regulation (McCrory et al., 2010; Watts-English et al., 2006). Indeed, there is a continuum of variability in children’s behavioral outcomes after experiences of chronic or extreme stress. While a growing literature suggests factors such as parental support and a high internal locus of control contribute to some of this variability (Masten, 2018b; Jaffee, 2017; Meng et al., 2018), relatively little is understood about how features of the early environment interact to shape children’s responses to stress (Smith and Pollak, 2021). A more comprehensive understanding of what factors modulate children’s responses to their environment can aid in the development of more targeted programs for children exposed to stress.

Self-regulation is broadly defined as the ongoing, dynamic, and adaptive modulation of internal mental states and/or behaviors to different contexts (Nigg, 2017; Pandey et al., 2018; Robson et al., 2020). This definition encompasses a range of different processes, including executive functioning, emotion and affect regulation, temperament, behavioral inhibition, self-control, impulsivity, and delay of gratification, among others (Diamond, 2013; Beauchaine and Zisner, 2017; Herman et al., 2017; de Ridder et al., 2012). Self-regulatory behaviors are multiply influenced behaviors characterized by an ability to inhibit or modify behaviors based on contextual demands (Weiner et al., 2015; Johansson et al., 2015). Behaviors in early childhood associated with a lack of self-regulation include externalizing behaviors, aggression, social withdrawal, inability to delay gratification, and impulsive behaviors (Weiner et al., 2015; Johansson et al., 2015; Baker, 2018). In children, poor self-regulatory behaviors can impede an individual from effectively navigating their social environment and have been linked to increased risk for mental and physical health problems later in life as well as poorer academic performance (Robson et al., 2020; Miller et al., 2018; Baker et al., 2019).

Stress refers broadly to demands placed upon an individual that require adaptation or change (McEwen, 2012; Sapolsky, 2015; McEwen and Akil, 2020). While stress is often beneficial, motivating individuals to respond effectively to threats and challenges in their environment, (McEwen, 2012) chronic, prolonged, or extreme stress (e.g., stress involving high levels of intensity and/or demands) has the potential to result in dysregulation of these responses via extended activation and disequilibrium of stress systems (Lupien et al., 2009; Smith and Pollak, 2020). Early life stress, or chronic, extended, or extreme stress occurring during childhood (Smith and Pollak, 2021), has been linked to a number of self-regulatory behaviors, including increased externalizing behaviors (Barch et al., 2017; Heleniak et al., 2016), conduct problems (Jaffee et al., 2009; Docherty et al., 2017), and anti-social behaviors (Shaw and Gilliam, 2000; Alink et al., 2012). This in turn places children at greater risk for psychopathology later in life (Palacios-Barrios and Hanson, 2019; Cloitre et al., 2008). However, not all children who experience early life stress demonstrate these later outcomes. To explain this variability, research examining the relationship between early life stress and children’s self-regulatory behaviors has primarily focused on whether a child has been exposed to an event or set of events predetermined by researchers to be stressors or adverse. Then potential moderators of children’s outcomes after event exposures are evaluated (McCrory et al., 2010; Masten, 2018a). Recent evidence indicates that approaches aimed at characterizing other features of the environment implicated in children’s perceptions of stress may aid in elucidating the mechanisms driving variability in children’s outcomes (Baram et al., 2012; Granger et al., 2020; Rivenbark et al., 2020; Danese and Widom, 2020).

One potential feature of interest is stability within the home environment. Instability within the home is associated with increased feelings of unpredictability and uncontrollability (Mccoy and Raver, 2014), and perceived control and predictability are two critical factors that contribute to variation in stress responses (Muller, 2012; Pruessner et al., 2005; Mormede et al., 1988). Factors such as number of people living in the home, number of caregivers, placement in foster care, and maltreatment exposure have all been linked to unpredictability and instability within the home and are associated with poorer self-regulatory behaviors (Hartman et al., 2018; Glynn et al., 2019). In addition, a stable school environment may act to counteract the effects of instability within the home environment. Indeed, many interventions targeting children in high stress environments represent school-based programs that emphasize high stability and predictability in addition to more specific intervention activities targeting social and emotional well-being (Domitrovich et al., 2007; Knight et al., 2019; Horn Ratner et al., 2006). To date, much of this research has examined two or three instability-related factors at a time and their relationships to children’s behavior. While this approach has provided insight into how specific factors may shape children’s development, it also represents only a small snapshot of children’s experience – capturing a few among many factors likely interacting to influencing children’s perceptions of predictability and controllability and experiences of stress (Smith and Pollak, 2021). Examining multiple environmental factors associated with stability is one avenue towards understanding how early environments influence variability in self-regulatory behaviors.

The current study used a model building approach to assess how multiple contextual factors associated with instability in the home influence self-regulatory behaviors in young children. This study was conducted in collaboration with a program that emphasizes consistent participant and predictable routines and activities targeting kindergarteners and preschoolers in high stress environments. This program is especially conducive to studying patterns of behavioral change because behaviors are documented at high frequency intervals and detailed information about children’s home environments is collected. We used this detailed information to identify children’s exposure to multiple environmental variables that have been posited to co-vary with instability in the environment. These included factors associated with experiences of severe or chronic stress, which is often characterized by high levels of unpredictability (Harms et al., 2018; Peters et al., 2017)—lifetime stress exposure, history of abuse, involvement in child protective services, and whether or not children were living with their biological parents or in adoptive/foster care—and factors that have been identified as indicators of high levels of environmental unpredictability and instability (Ellis et al., 2009; Li et al., 2023)—number of residential moves, number of people living in the home, and number of changes in primary care source. Together, these data allowed us to examine changes in children’s self-regulatory behaviors at a high sampling rate and how interactions across multiple sources of potential instability along with the introduction of a stable environment (the school program) influence these changes.

## Materials and methods

### Participants

Participants were 136 children ages 3 to 7 years (*M* = 4.95, *SD* = 0.75; 99 male; 46.3% children were White, 31.6% African American—Black, 5.2% Hispanic, and 16.9% Multi-Racial) who participated in a program located in Elkhart, Indiana which enrolls children who have experienced some form of or have been identified as at-risk of exposure to early life stress. Data were available for all children who participated in the program between 2006 and 2016. 24.3% of these children were no longer living with their biological parents (i.e., in foster care or placed in guardianship) when they started the preschool program (see Supplementary Table S1 for placement information). On average, children were in the program for 6.47 months (SD = 3.31 months; Range: 1–19 months). This study was approved by both the Child and Parent Services (CAPS) Building Block’s Program and the University of Chicago Institutional Review Board and was conducted in accordance with relevant guidelines and regulations. All parents of participants in the program provided written informed consent with the school on their child’s entry into the program for use of their child’s data.

### Preschool program

#### Program overview

The CAPS Building Block’s Program enrolls preschoolers and kindergarteners at risk of early life stress and behavioral problems. Students are referred to the program by the public school system, child protective services, and other child service agencies (mental health, social work, etc.). Reasons for referral included severe behavior issues (*n* = 105), potential experiences of maltreatment (*n* = 19), exposure to domestic violence (*n* = 4), other family environment concerns (e.g., family member drug use, *n* = 5), organizational referral (Headstart, Healthy Families) with reason unknown (*n* = 2), or unknown (*n* = 1). The school uses a rolling admissions model, with students admitted throughout the year, starting in late August, and gradually transitioned into another classroom after a determination is made by school staff that the child is ready to transition. The school has a small child-to-teacher ratio (three teachers for one classroom of less than 20 children) and uses an individual-based approach, with teachers adjusting their teaching techniques based on the child and their specific situation. This allows teachers to spend time one-on-one with children to support their use of self-regulatory techniques. Time outs and safe holds (a safe form of restraining a child until they are calm when their behavior becomes dangerous to themselves and/or others in the classroom) are used when children are displaying disruptive negative behaviors in the classroom.

An emphasis of the program is the implementation of a predictable and consistent school environment, reflected in highly structured and consistent day to day activities, predictable and reliable consequences for behaviors, and a requirement that participants are attending and engaging in all program activities. This is reflected in the relatively low rate of absences (*M* = 0.12, *SD* = 0.09). The school works with both the children and families. All caregivers are required to participate in parenting classes and weekly in-home visits provided by preschool staff. Teachers in the classroom document classroom behavior and events, including monthly ratings of children’s self-regulatory behaviors and documenting the number of time outs and safe holds a child is placed in each day.

#### Child enrollment, admission, and program transition

An entry coordinator staff member interviews each prospective child and family and assesses risk prior to enrollment. Children are primarily admitted on a first come first serve basis from the referrals, with the preschool aiming to be a last resort for service – i.e. if families have not pursued alternative options for support the preschool will refer them to those prior to allowing a child admission. Additionally, families of children with identified developmental disabilities or clinical disorders are referred to alternative services in the area. A transition out of the program is based on teacher observation of child behaviors both at the preschool and at the other school or completion of the program at the end of the academic year in early June.

#### Activities supporting self-regulation

Teachers help support the use of a variety of self-regulatory behaviors in children, including emotion recognition (i.e., teaching the child to identify their own and others’ emotions), appropriate emotion expression techniques (i.e., children are giving strategies through which they can express their emotions such as “stomping out their mad” instead of screaming, hitting, scratching etc.), and appropriate peer interaction and play (i.e., teachers work with children to prevent hitting, scratching, biting and other forms of violent behavior in peer interactions), as well as typical academic skills including reading, writing, and math. In addition to the reinforcement tools described in the manuscript, teachers also use positive reinforcement tools such as providing children with stickers or stamps when children are able to effectively utilize the tools they have been taught, manage to avoid conflict, participate in a task as requested, and so on.

#### Caregiver components

All caregivers (parents or guardians) of children enrolled in the preschool program are required to complete parenting classes, either through classes offered at CAPS or other community classes, as well as weekly in-home visits, provided by preschool staff. In these visits, the home visitor works with parents to help implement effective parenting strategies and techniques in the home, including discouraging the use of corporal punishment, teaching parents to implement and follow through logical consequences using techniques such as time outs to discipline their children, and teaching parents to encourage similar forms of emotion expression (i.e., “stomping out their mad” or “shaking out their sad” instead of hitting or kicking) as those used in the classroom in their children at home. Additionally, the home visitor, a staff member trained by the preschool, acts as another source of support for these struggling families by providing parents with someone they can talk to about issues in the home and helping connect parents to available resources (healthcare, childcare, etc.).

#### Procedure and measures

As part of the school program, staff extensively document information about the child’s home and family environment and past history of stressors. Teachers in the classroom document classroom behavior and events, including monthly ratings of children’s self-regulatory behaviors. Included in these documents are daily qualitative records of child behavior (beyond teacher ratings). Because teachers spend one-on-one time with the children, they are able to track and note subtle changes in behaviors, both positive and negative, in these daily summaries. This detailed, high frequency documentation is used by teachers to inform the monthly ratings of self-regulatory behaviors. The current study focuses on two aspects of data from information documented by program staff: (1) Measures of children’s self-regulatory behaviors from teacher documentation of children’s behavior in the classroom; and (2) Measures of environmental instability at both the level of the home and school participation coded from school documentation of the child’s home and family environment and past history of stressors.

### Child self-regulatory behaviors

#### Teacher ratings

Teachers are trained to complete monthly ratings of children’s ability to cope with disappointment, express anger appropriately, and control impulses on a Likert scale from 0 to 4, with higher numbers indicating more effective self-regulation (see Supplementary materials). Ratings were modified from the Devereux Early Childhood Assessment (DECA) Scale (LeBuffe and Naglieri, 1999; Lien and Carlson, 2009). Items were selected to be capture commonly assessed domains of self-regulation in standardized rating measures for education settings (see Ross and Ascetta, 2024) while also not placing an excessive demand on teachers. All items were highly correlated (Supplementary Table S2) and summed to form a composite teacher rating of child self-regulatory behavior for each month. Composite ratings demonstrated an intraclass coefficient (ICC) of 0.65, indicative of moderate test–retest reliability similar to other widely used measures (Howard et al., 2022; Reilly and Downer, 2019). Cronbach’s alphas at each time point were > 0.80 suggesting high levels of internal reliability.

#### Environmental instability

Prior to children entering the program, the school performed a structured intake interview with parents/guardians in which they asked a variety of questions about the child’s home and family structure as well as past history of stress. Variables coded from this data to examine potential environmental influences on child self-regulatory behavior and behavioral changes during the program were: (1) *Number of people in home:* the total number of people currently living in the home (adults and children) was calculated as a sum of adults and children in the household, (2) *Living in biological care:* whether a child was living with their biological parents or in an alternative care situation like adoptive parents, foster parents, or in guardianship at the start of the program was coded as a binary categorical variable (biological parents/other). As the majority of children not living with their biological parents were in foster care (69.7%, Supplementary Table S1) we did not attempt to examine specific effects of type of alternative care, (3) *Parent configuration*: parent configuration was coded as a binary categorical variable of single parent or two parent household, (4) *Lifetime stress exposure:* child’s exposure to prior stress was calculated as a sum score of the number of stressors in the family a caregiver marked on a lifetime stressors checklist (Supplementary materials), a standard method for characterizing cumulative lifetime stress using checklist measures (Felitti et al., 1998; Sarason et al., 1978), (5) *History of abuse:* parent/guardian reported past abuse of the child (“Has the child experienced or witnessed abuse?”) was coded as binary categorical variable (abuse/no abuse), Three additional factors documented throughout the school year were also used to examine environmental instability: (1) *CPS Involvement*: school documentation of number of child protective services (CPS) reports filed during the program were coded as a sum score of number of reports filed by teachers or school staff while the child was in the program (for more information see Supplementary materials), (2) *Number of moves:* The total number of times a child’s family moved homes during their participation in the program was calculated as a sum of moves that occurred while the child was enrolled in the school program, (3) *Number of primary care source changes:* If a child changed care source (i.e., moving from care with biological parents to guardianship or foster care), this was coded and calculated as a sum of changes in care sources that occurred while the child was enrolled in the school program. These variables were identified as they have been characterized as important indicators in household chaos and stability in previous research (Glynn et al., 2019; Belsky et al., 2012; Vernon-Feagans et al., 2016; Evans and Wachs, 2010). Correlations between study measures can be found in Supplementary Table S2 and on the Open Science Framework (OSF)^1^.

### Statistical analyses

**Figure 1:**
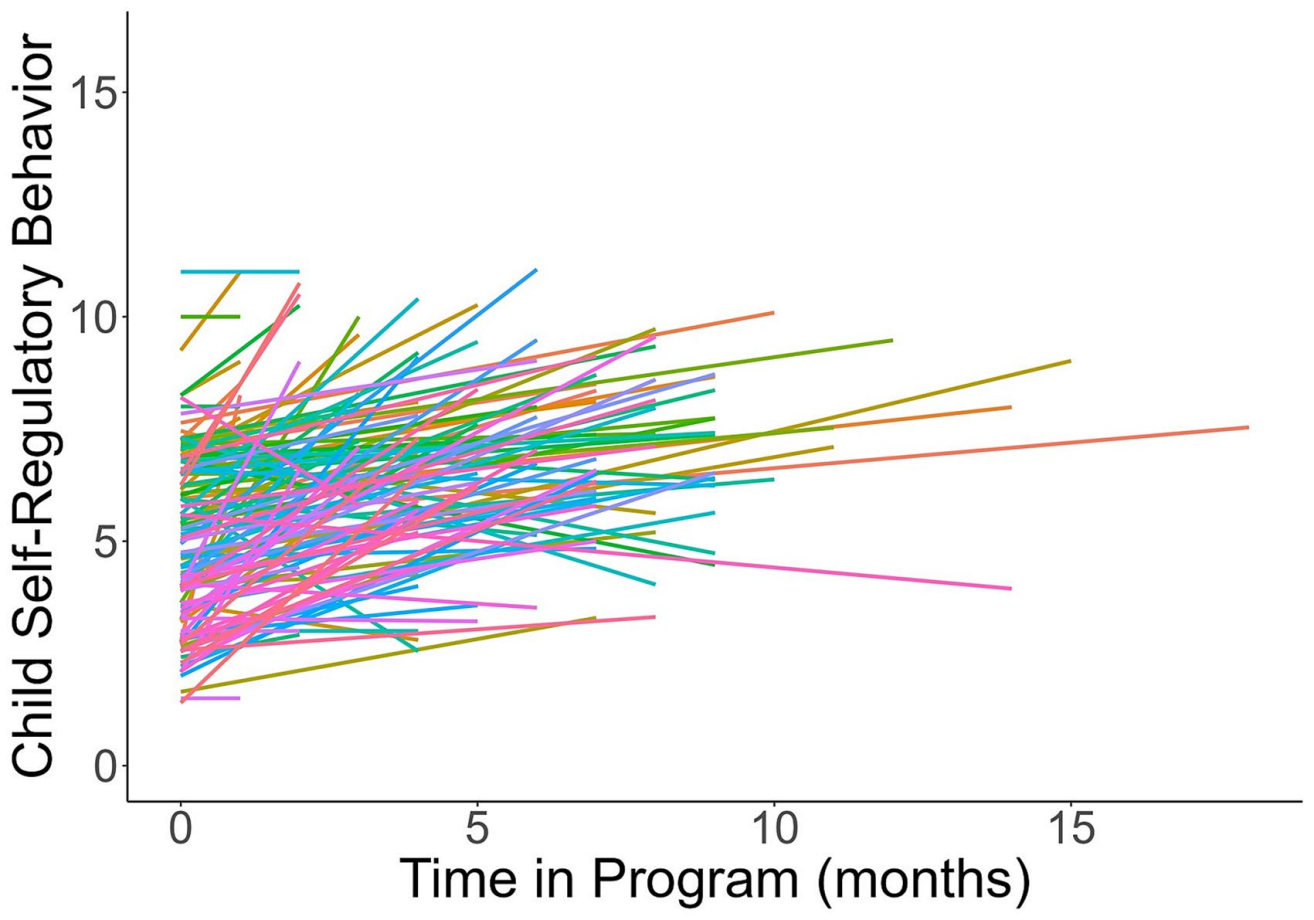
Subject level trajectories of changes in teacher-rate self-regulatory behaviors during the program.

Descriptives for study variables are in Figure 1 and Table 1. To assess changes in children’s self-regulatory processes over time, we utilized longitudinal hierarchical linear modeling (HLM) techniques (see Supplementary materials; Singer and Willett, 2003). All models were run using the nlme package (Pinheiro et al., 1999) for R (v4.3.0; R Core Team, 2023) with full maximum likelihood estimation in R Studio (Posit Team, 2023). Inspection of individual subject level trajectories for teacher ratings indicated that a linear growth model was most appropriate for the data set. In the models, time was treated as random and nested within subject. To better elucidate what factors play a role in changes in children’s self-regulatory behaviors, we took a model building approach to our analyses. Following recommended model building techniques (Singer and Willett, 2003), an initial model, including only fixed and random effects of time in the program to examine general patterns of growth for each outcome measure. To account for the fact that children entered the program on a rolling basis, time was coded such that each child’s initial month in program was 0. Treating the initial month as 0 means intercept values convey information about children’s behavior upon their entry to the program, and time effects represent changes since starting the program. Environmental predictors were then incorporated in a stepwise manner into the models as fixed predictors at the subject level to examine influences of these factors on children’s self-regulatory behaviors and changes in these behaviors over time. These models were compared using Log Likelihood (LL), Akaike Information Criterion (AIC), and Bayesian Information Criterion (BIC) statistics to determine which model best fit the data (Singer and Willett, 2003; Raudenbush and Bryk, 2002).

**Table 1:** Descriptives for all variables at the start of the program.

	Full sample (*n* = 136)			Best fit model sample (*n* = 119)		
	Mean	SD	Range	Mean	SD	Range
Age (years)	4.95	0.75	3–7	4.91	0.70	3–6
Prior exposures to stress	3.09	2.27	0–10	3.09	2.24	0–10
Number of people in house	3.88	1.76	1–13	3.85	1.78	1–13
Number of CPS reports	0.37	1.00	0–7	0.40	1.05	0–7
Number of moves	0.36	0.66	0–3	0.36	0.63	0–3
Number of primary care source changes	0.13	0.44	0–3	0.12	0.37	0–2
Proportion of absences (out of total school days)	0.12	0.09	0–0.5	0.11	0.09	0–0.36
Months in program	6.47	3.32	1–19	6.58	3.37	1–19

		N (%)	N (%)
Gender	Male	99 (72.8)	85 (71.4)
	Female	37 (27.2)	34 (28.6)
Living with biological parent(s)	Biological parents	102 (75.0)	91 (76.5)
	Non-biological parents	33 (24.3)	28 (23.5)
	Information not available	1 (0.7)	0 (0)
Family configuration	Two parent	42 (30.9)	37 (31.1)
	Single parent	94 (69.1)	82 (68.9)
History of abuse	Abuse	43 (31.6)	41 (34.5)
	No abuse	86 (63.2)	78 (65.6)
	Not reported	7 (5.1)	0 (0)

HLM can handle missing values for the repeated measures using maximum likelihood estimation (Singer and Willett, 2003; Raudenbush and Bryk, 2002) assuming data is missing at random (MAR). To confirm MAR, we examined correlations of number of time points with outcome data with other model covariates and found no evidence of strong associations (*p*s > 0.10) with the exception of age, such that older children had fewer time points (*r* = −0.34, *p* < 0.001). We also examined whether there was any systematic variation in missing data at the level of the covariates. With the exception of number of people living in the home (*t*(6.07) = −3.02, *p* = 0.02) and number of CPS reports filed (*t*(55.05) = 2.04, *p* = 0.05), there were no differences in any of the covariates included in the models. While these differences may be indicative of missingness not being random, it would likely be due to covariate dependent dropout (CDD), or dropout that is dependent on predictors included in the model. CDD allows for associations between the probability of missing values and observed substantive predictors, as long as it is unrelated to the contemporaneous value of the associated outcome (Singer and Willett, 2003; Laird, 1988; Bartlett et al., 2014; White and Carlin, 2010). Therefore, analyses including covariates were run using complete cases only.

We ran all final models controlling for age, gender, race and teacher who did the ratings (for teacher ratings of self-regulatory behaviors), including them as fixed covariates, to ensure these factors did not influence any findings. Both race and teacher were dummy coded. For race, the comparison category was White. For teacher, the comparison group was the teacher who had been in the program the longest. To better understand and interpret any significant interactions observed, simple slopes were computed following recommendations by Preacher et al. (2006) using package emmeans (Lenth, 2019). A saturated model including all predictors and covariates is included in Supplementary Table S3. Our goal was to examine how indices of instability in the home environment contribute to children’s trajectories of self-regulatory behaviors, and thus we focused on these factors as predictors of behavior rather than outcome variables. For all models reported in the manuscript, we examined the residuals to ensure they did not violate assumptions of normality and homogeneity of variance. There was minimal evidence for strong violations. All residual plots can be found on OSF (see footnote 1).

## Results

### Self-regulatory behaviors over time

In the unconditional growth model (*AIC =* 2906.28, *BIC* = 2934.90, *LL =* −1447.14, *r^2^* = 0.18; *n* = 135), which included only time in the program as a predictor, there was a significant positive effect of time on children’s teacher-rated self-regulatory behavior (*β* = 0.33, *SE* = 0.03, *p* < 0.001). Including age, gender, race, and teacher (for teacher ratings) did not change the effects.

### Effects of home environment on teacher-rated self-regulatory behavior

**Figure 2:**
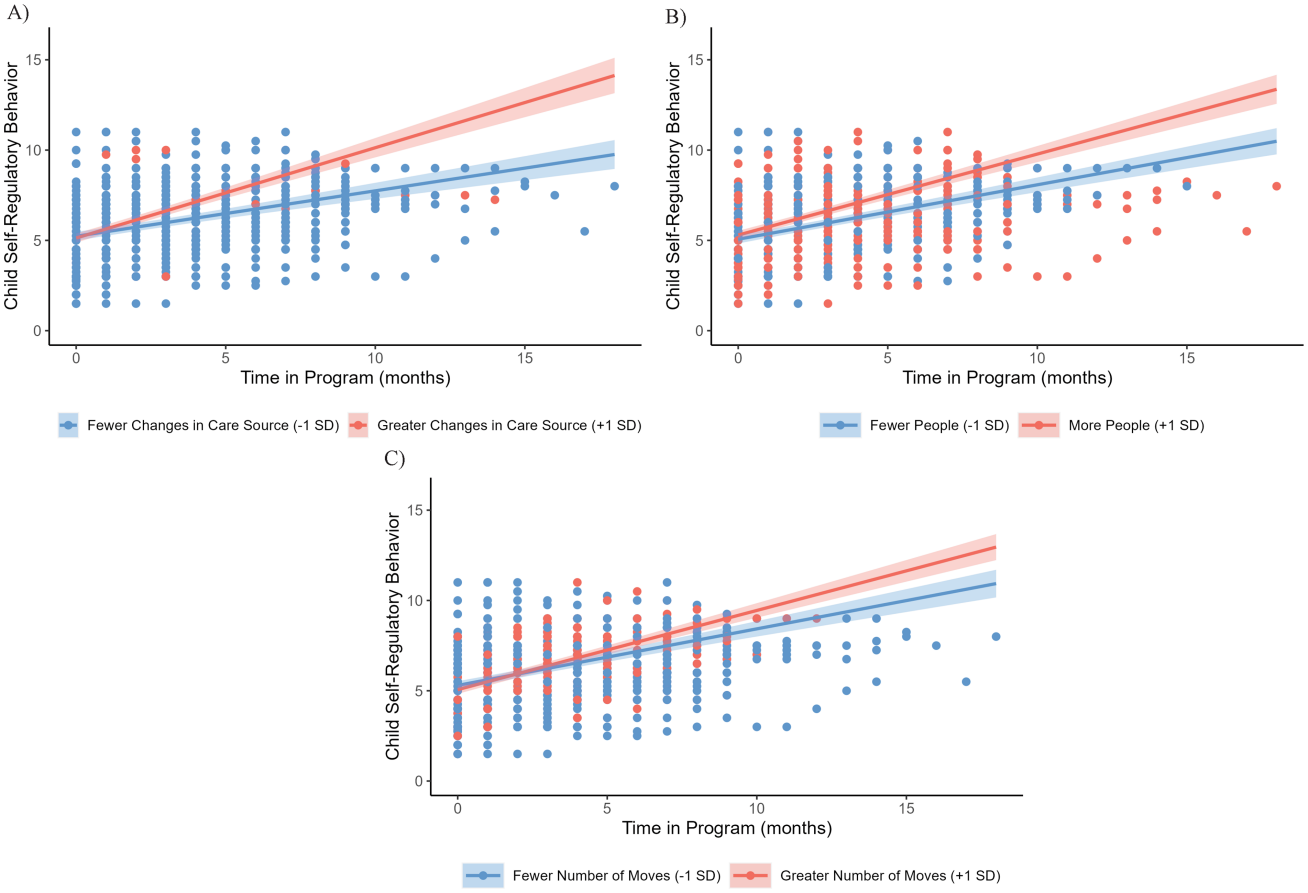
**(A)** Relationship between number of changes in primary care source and changes in teacher-rated self-regulatory behaviors during the program. **(B)** Relationship between number of people living in the home, time in the program and teacher-rated self-regulatory behaviors. **(C)** Relationship between number of moves, time in the program, and teacher-rated self-regulatory behavior at the beginning of the program. Shading represents the confidence band. Data points are colored based on whether 1 standard deviation (red) or below the mean (blue).

The model which best fit the data (*AIC* = 2606.32, *BIC* = 2708.91, *LL* = −1281.16, *r^2^* = 0.28; *n* = 119; Table 2) included time, number of people living in the house, living with biological parent(s), lifetime stress exposure, number of moves, number of CPS reports, number of primary care source changes, and the following interactions: (1) between stress and number of moves; (2) between number of primary care source changes and number of CPS reports while in the program. The positive effect of time on children’s teacher-rated self-regulatory behavior remained significant (*β* = 0.38, *SE* = 0.03, *p* < 0.001). There was also a main effect of children living with their biological parent(s) (*β* = 0.98, *SE* = 0.41, *p* = 0.02). Children living with their biological parents at the start of the program had lower teacher ratings of self-regulatory behavior their first month in the program compared to children not living with their biological parent(s). There was a significant interaction of primary care source changes with time in the program (*β* = 0.31, *SE* = 0.11, *p* < 0.01), such that children with a greater number of primary care source changes (+1SD) demonstrated greater positive change during the program in teacher-rated self-regulatory behaviors (*β* = 0.50, *SE* = 0.06, *p* < 0.001) than children with fewer primary care source changes (−1SD) (*β* = 0.25, *SE* = 0.05, *p* < 0.001) (Figure 2A). Number of people living in the house (*β* = 0.04, *SE* = 0.02, *p* = 0.03; Figure 2B) and number of moves also interacted with time (*β* = 0.10, *SE* = 0.05, *p* = 0.04; Figure 2C). Children living with more people in the house (+1SD) demonstrated increased positive change in teacher rated self-regulatory behaviors during the program (*β* = 0.45, *SE* = 0.05, *p* < 0.001) as compared with children living with fewer people in the house (−1SD; *β* = 0.30, *SE* = 0.04, *p* < 0.001). Children who moved more frequently (+1SD) also demonstrated increased positive change in teacher rated self-regulatory behaviors (*β* = 0.44, *SE* = 0.04, *p* < 0.001) as compared to those who moved less frequently (−1SD; *β* = 0.31, *SE* = 0.05, *p* < 0.001).

**Table 2:** Effects for best fit model.

**Fixed Effect**	**β (SE)**	**df**
Intercept	5.20 (0.16)***	655
Time	0.38 (0.03)***	655
Living with biological parent(s)	0.98 (0.41)*	110
Number of people in house	0.06 (0.09)	110
Number of CPS reports	-0.32 (0.18)^†^	110
Prior exposure to stress	0.08 (0.08)	110
Number of moves	-0.18 (0.26)	110
Number of primary care source changes	-0.11 (0.49)	110
Living with biological parent(s)*Time	-0.13 (0.08)^†^	655
Number of people in house*Time	0.04 (0.02)*	655
Number of CPS reports*Time	0.05 (0.04)	655
Prior exposure to stress*Time	0.02 (0.01)^†^	655
Number of moves*Time	0.10 (0.05)*	655
Number of primary care source changes*Time	0.31 (0.11)**	655
Number of moves*Prior exposure to stress	-0.35 (0.14)*	110
Number of moves*Prior exposure to stress*Time	0.09 (0.03)***	655
Number of CPS reports*Number of primary care source changes	-0.62 (0.88)	110
Number of CPS reports*Number of primary care source changes*Time	0.80 (0.23)***	655
Random Effects	Variance	
Intercept	2.33	
Time	0.06	
Model fit statistics		
AIC	2606.32	
BIC	2798.91	
Log Likelihood	-1281.16	
r^2^	0.28	

^†^indicates *p* < 0.10; *indicates *p* < 0.05; **indicates *p* < 0.01, ***indicates *p* < 0.001.

**Figure 3:**
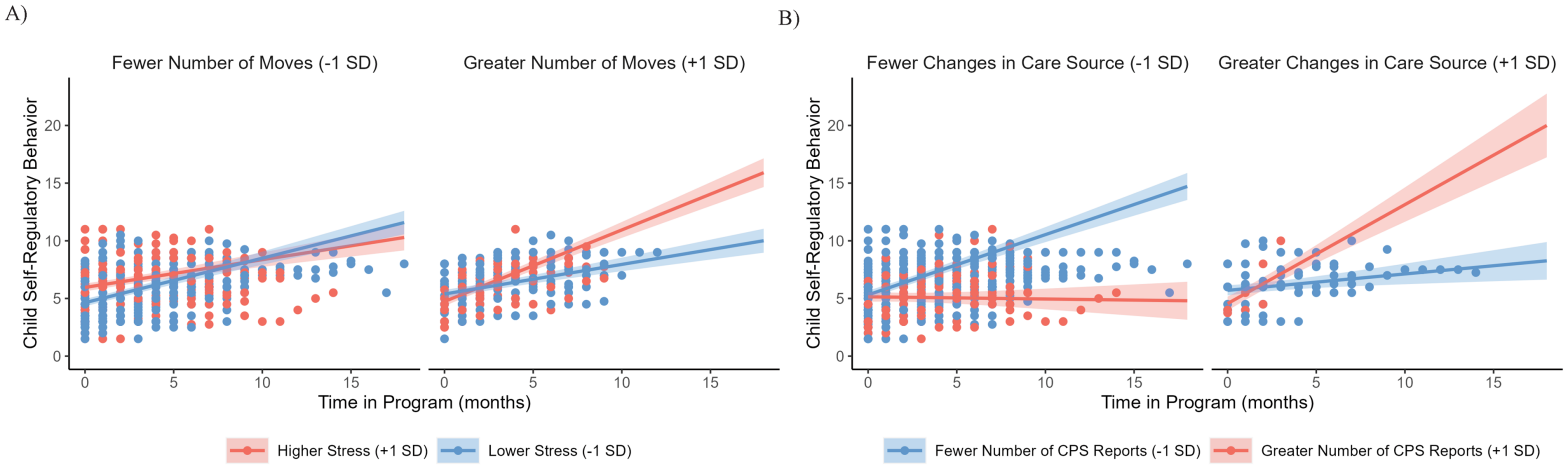
**(A)** Relationship between lifetime stress exposure, number of moves, and change in teacher-rated self-regulatory behaviors during the school program. **(B)** Relationship between total CPS reports, changes in primary care source, and change in teacher-rated self-regulatory behaviors during the school program. Shading represents the confidence band. Data points are colored based on whether 1 standard deviation above (red) or below the mean (blue).

There was a significant interaction of lifetime stress exposure with number of moves (*β* = −0.35, *SE* = 0.14, *p* = 0.01). Comparisons of the simple slopes for number of moves and lifetime stress exposure indicated that at the beginning of the school program for children with higher levels of stress (assessed at 1 SD above the mean), number of moves demonstrated a negative association with self-regulatory behaviors (*β* = −0.96, *SE* = 0.43, *p* = 0.03). At lower levels of stress (assessed at 1 SD below the mean) the association of number of moves with self-regulatory behaviors was not significantly different from zero (*β* = 0.59, *SE* = 0.38, *p* = 0.12). Additionally, lifetime stress exposure and number of moves demonstrated a significant interaction with time (*β* = 0.09, *SE* = 0.03, *p* = 0.001; Figure 3A). Examining the simple slopes indicated that for children who experience a greater number of moves, higher levels of lifetime stress exposure was associated with increases in self-regulatory behaviors over time in the program (*β* = 0.62, *SE* = 0.08, *p* < 0.001) compared to children with lower levels of lifetime stress exposure (*β* = 0.26, *SE* = 0.06, *p* < 0.001). Among those with fewer moves, differences between higher levels (*β* = 0.24, *SE* = 0.07, *p* < 0.001) and lower levels (*β* = 0.39, *SE* = 0.06, *p* < 0.001) of lifetime stress exposure were smaller and in the opposite direction. There was also a significant interaction between number of CPS reports, number of primary care source moves, and time in the program (*β* = 0.80, *SE* = 0.23, *p* < 0.001; Figure 3B). Examining the simple slopes indicated that for children who experienced a greater number of primary care source moves (+1SD), children with a greater number of CPS reports (+1SD) while they were in the program demonstrated a significant positive effect of time in the program on teacher-rated self-regulatory behaviors (*β* = 0.86, *SE* = 0.16, *p* < 0.001) while children with fewer CPS reports (−1SD) did not (*β* = 0.14, *SE* = 0.10, *p* = 0.14). In contrast, children with fewer primary care source moves (−1SD) demonstrated the opposite effect, with children with fewer CPS reports while they were in the program demonstrating a significant positive effect of time in the program on teacher-rated self-regulatory behaviors (*β* = 0.52, *SE* = 0.07, *p* < 0.001) while children with a greater number of CPS reports did not (*β* = −0.02, *SE* = 0.10, *p* = 0.85). Including these interactions improved model fit (*p* < 0.001). Including age, gender, race, and teacher resulted in the interaction between number of moves and time in the program becoming non-significant (*β* = 0.09, *SE* = 0.05, *p* = 0.06), but did not change any of the other observed effects.

### Sensitivity analyses

We ran additional analyses to determine reported effects were robust. To ensure estimates were not biased due to children having very few time points (< 3; see Singer and Willett, 2003) or being in the program for more than one academic year (~ 10 months), we reran all models dropping those subjects with less than three time points (*n* = 14) and data for timepoints greater than 10 months. All effects remained comparable. Additionally, to ensure models, particularly the three way interactions, were not biased by a few children with higher numbers of CPS reports, moves, and changes in care source (see Supplementary Table S4 for cross-tabulation for these covariates), we reran all models treating those variables as binary (none or present). Again, effects remained comparable. Given no *a priori* power analysis was conducted due to the limitations of working with existing data collected in a naturalistic setting, we conducted sensitivity analyses using a Monte Carlo simulation approach to probe power of observed significant effects (DeBruine and Barr, 2021). Simulations (*n* = 1,000) estimating power for the observed effect sizes and a range of effects both smaller and larger were run. These analyses suggested all effects demonstrated power at or above 0.80, with the exception of the interaction between lifetime stress exposure and number of moves (power > 0.75). Full model output for all additional analyses can be found on OSF (see footnote 1).

## Discussion

The goal of this study was to identify environmental factors, using multiple measures of instability in the home and school environment, that contribute to patterns of self-regulatory behavior change in children exposed to early life stress. We examined how indices of environmental instability interact and affect children’s self-regulatory behavior changes. We identified several environmental factors that contributed to patterns of self-regulatory behaviors, including children’s care source, changes in care source, lifetime stress exposure, CPS involvement, and number of family moves. Over the course of the program, we observed children’s self-regulatory behaviors increasing. Together these data suggest that children at higher risk of stress exposure and problematic self-regulatory behaviors benefit from involvement in a stable and reliable environment (i.e., the school) and this benefit is greatest for children in environments characterized by more instability.

Children with higher levels of lifetime stress exposure and a greater number of moves during the program demonstrated the greatest levels of positive self-regulatory behavior change. Additionally, children who experienced more changes in their primary care source demonstrated more positive change in teacher-rated self-regulatory behaviors during the program. This effect was most pronounced for children who had more CPS reports filed while they were in the program. Higher stress exposure, more household moves, lack of stability in care placement, and greater involvement with CPS have all been linked to increased instability and unpredictability within the home environment (Belsky et al., 2012; Gerin et al., 2017; Rubin et al., 2007). Together, this suggests that children in environments typically characterized as unstable demonstrated the most pronounced positive change in self-regulatory behaviors after the implementation of a stable environment, in this case, the school.

We also found that populations traditionally considered as at greater risk for problematic self-regulatory behaviors (children in foster care/guardianship placement) (Newton et al., 2000) demonstrated better self-regulatory behaviors in their first month in the program as compared to those living with their biological parent(s). One potential explanation for these findings is that children who have been removed from their biological parents’ care have greater experience with change and disruption and are better able to adjust to beginning the school program. This explanation is in line with evidence suggesting children who experience higher levels of caregiver instability early in life demonstrate increased behavioral flexibility (Fields et al., 2021) and enhanced cognitive performance in uncertain environments (Young et al., 2018). Alternatively, children who have been moved out of their biological parents’ care may now be in more stable home environments. However, this seems less likely given evidence suggesting out of home placements are associated with high levels of instability (Fisher et al., 2013; Leathers et al., 2019). Our findings related to changes in primary care source suggest it is possible that the variability in a child’s care sources may be a more important influence on behavior change than initial care source. Further examining questions related to variability in care and placement structure could help provide insight into what may be driving these findings. For example, it is possible that children who have more recently moved care may demonstrate an initial period of stability that is then followed by a relapse in behaviors. These questions should be explored in more depth to better understand differences between children living with their biological parents and children no longer in the care of their biological parents.

Last we found having a greater number of people living at home was associated with increased positive change in teacher rated self-regulatory behaviors over the course of the program in the first month of the program. This is somewhat inconsistent with previous literature. Number of people living in the household is often included as a measure of household instability and has been associated with poorer self-regulatory behaviors (Vernon-Feagans et al., 2016; Berry et al., 2016). It is possible that this discrepancy is due to consistency of the number of people in the home (i.e., are people moving in and out of the home), support or lack of support children receive from those relationships, and children’s perceptions of those relationship as consistent rather than the number of people in the home. Indeed a growing literature indicates that children’s perceptions of their parental and peer relationships play an important role in influencing children’s self-regulatory outcomes (Michaelson and Munakata, 2020; Brody et al., 2019; Brody et al., 2016).

The current research has several strengths, primary being that it utilizes a data set that would be difficult to replicate within the laboratory, in which a wealth of data is documented in both the classroom and home environment. This richness in data provides access to information about a wide range of factors that may contribute to how children perceive and interpret their early environments. This type of research, which examines multiple environmental indices in concert is necessary to better characterize how different aspects of the environment influence development—currently, research relies on a small set of events researchers have identified as representing salient aspects of the early environment. However, event based approaches have demonstrated limited utility in explaining individual differences in children’s outcomes (Smith and Pollak, 2021). Collaborating with the school allowed us to utilize a unique source of data which is typically difficult to obtain, consisting of a range information on environmental experiences and children’s self-regulatory behaviors sampled at a high frequency. This access allowed us to examine how different common indicators of environmental stability influence children’s patterns of self-regulatory behavioral change. In order to advance our understanding of children’s development and the effects of early environments on development, it is critical more research aimed at characterizing the environment utilizing multiple indices of environmental exposure at the school and family level be conducted.

Despite these strengths, there are several limitations of this study that should be mentioned. The primary limitations are linked to working within the structures for data collection already in place in the context of the school program. Among these are the fact the school program itself is oriented towards supporting children in high-risk environments, which makes it difficult to disentangle whether findings generalize outside of this population and participation in this program. However, this can also be considered a strength, as conducting the study in the context of the program allowed us to examine how multiple environmental indices associated with instability interact with the implementation of a more stable environment (via the school program) to shape self-regulatory behaviors. The fact data collection occurred in the context of an existing school program also means the sample consisted primarily of children experiencing severe behavioral difficulties. This fact limits generalizability to other populations and means that it is possible changes in self-regulation during the program may influence changes in the environment (reduced CPS reports, increased likelihood of living outside of the home). Future research collecting measures of self-regulation prior to entry in similar programs could help alleviate these concerns.

An additional limitation of the data set is its reliance on teacher reported child behaviors. The scale implemented was selected by the school program to collect critical data while also not increasing teacher burden. Therefore, the selected scale does not capture all possible domains of self-regulation. Indeed, the construct of self-regulation is broad, and there is continued debate on both what the construct represents and how to best assess it (Diamond, 2013; Ross and Ascetta, 2024). Our results should be interpreted in the context of the domains captured by the current items. Future research using multimodal assessment is necessary to disentangle whether these results are comparable for other domains of self-regulatory processes.

It is also possible that the sources of measurement introduced different types of bias. It may be that the positive change in ratings represents teachers and children becoming more attuned to each other. Teachers are not blind to children’s home situation and typically are familiar with the child’s home environment prior to the child starting the school; it is possible teachers may rate children differently based on their knowledge about the home environment. However, teachers are not instructed to treat different groups of children differently as they enter the program and are trained to utilize similar rating and teaching techniques based on children’s behavior in the classroom. While teachers may be rating children more positively out of a desire to demonstrate themselves to be an effective teacher, this seems unlikely given that despite there is an overall positive pattern of change, there is a range of variability in these ratings both across individuals and across time. Teacher report is a common assessment tool for self-regulatory behaviors in education settings and the ratings employed in the current study assessed domains comparable to those of widely used standardized rating scales for children’s self-regulatory behaviors (Ross and Ascetta, 2024). Our indices of environment instability also varied in the source of the report (e.g., CPS as compared to parent report). These different types of reports could have introduced different sources of measurement bias in our predictor (Cooley and Jackson, 2022; McTavish et al., 2020). Future work collecting multiple measures of potential sources of instability and child behaviors can provide increased insight into how to best measure and assess children’s environments.

Last, the current findings are somewhat limited by the sample being predominantly male and the sample size relative to the number of tests. Research suggests that girls demonstrate increased and earlier emergence of self-regulatory behaviors (Montroy et al., 2016; Broekhuizen et al., 2015; Coyne et al., 2015). Future research should assess whether the findings hold for a larger sample with more females using more standardized measures of self-regulation. Additionally, the focus of the current research was on how child characteristics influence patterns of behavioral change but future research can examine whether teacher characteristics, like race, age, or experience play a role in children’s outcomes. Despite these limitations, the current research represents a critical initial step in examining how multiple factors within a naturalistic setting interact to shape children’s self-regulatory behaviors, that can be used to inform more controlled laboratory studies.

Future research can expand on the current findings by examining the potential mechanisms through which instability in the environment influences children’s self-regulatory behaviors. One potential mechanism for the observed relationships between stability and self-regulatory behaviors is that the predictability associated with a more stable environment leads to increased feelings of safety and security. Safety and security are thought to be important to engaging and scaffolding the development of prefrontal circuits, particularly the ventromedial prefrontal cortex, that play a critical role in self-regulation (Porges, 2015; Gunnar et al., 2015). Alternatively, some theories of self-regulation, especially those linked to self-control, propose the ability to self-regulate is linked to the coping resources an individual has available to them (de Ridder et al., 2012; Muraven and Baumeister, 2000). It is possible in more stable environments the coping demands placed on children are reduced, resulting in them having increased resources available for self-regulation. These explanations are not mutually exclusive and can be experimentally tested in future studies. Additionally, it is the case that some of the environmental indices may not always equate to increased instability or perceived unpredictability. We focused on these measures as they are those which have been theorized and utilized as indices of instability in previous research (Hartman et al., 2018; Glynn et al., 2019). However, examining more concretely how they relate to children’s perceptions of stress and predictability can aid in illuminating these relationships.

Overall, this study suggests that there are important individual differences in patterns of self-regulatory behavior change in children exposed to early life stress. It points to complex interactions between children’s home environment, implementation of a more reliable and stable environment, and changes in self-regulatory behavior. Our findings that children from environments characterized by markers of higher levels of instability demonstrate the most pronounced positive behavior change suggest these children may be the ones most benefitted by interventions aimed at increasing stability and consistency. Future work should focus on the mechanisms driving these complex relationships to both better understand how instability shapes development and identify the most promising targets for intervention. Work oriented towards identifying what drives these differences in children’s patterns of self-regulatory behavioral change and the role children’s perceptions of their early environment plays in these relationships can inform who may be most vulnerable to certain environments as well as what factors reduce the likelihood of a child experiencing an event or environment as stressful. Together, this can help researchers and clinicians better target programs for specific subpopulations of at-risk families and children.

## Data Availability

The datasets presented in this study can be found in online repositories. The names of the repository/repositories and accession number(s) can be found at: https://osf.io/y3u4q/.

## Footnotes

^1^ https://osf.io/y3u4q/
