# Supplementary material for: Instability in the environment and children’s in-school self-regulatory behaviors
Source: Front Psychol. 2025 Mar 18;16:1498961. doi: 10.3389/fpsyg.2025.1498961 (PMC11958713; doi:10.3389/fpsyg.2025.1498961)
Supplement: Supplementary file 1 [file Supplementary_file_1.docx]

Supplemental Materials

**Lifetime Stressors Checklist**

Please check those events that have occurred in the child’s family*:

- Divorce
- Financial Hardship
- Inadequate Housing
- New Baby
- Death of a Loved One
- Mental Health Issues in Immediate Family
- Child Abuse in Family**
- Spousal Abuse
- Alcohol or Drug Use
- Parent in Prison
- Child or Sibling Lived Out of the Home

With Who: _______________ How Long: _______________

*Preschool staff conducting interview emphasized that the focus should be on events occurring within the child’s time in the family

**It is possible there is some overlap between this item on the lifetime stressors checklist and the abuse of child predictor in the models. However, a response of yes to this item is not specific to the child enrolled and could represent abuse of any child in the family. Given a separate question asked specifically about abuse of the child enrolled in the program, we elected to keep this item with the lifetime stressors checklist. Running models with and without the item produced similar results.

**Teacher Rating Scale**

Rated using a scale from 0 – 4 with 0 being never and 4 being always

1. Child expresses anger appropriately
2. Child copes with disappointment or change
3. Child controls impulses

Items were summed to form a composite score of children’s teacher rated self-regulatory behaviors

**Additional Information on Measures of Self-Regulatory Behaviors**

Teacher ratings of children’s self-regulatory behaviors are based on children’s ability to respond using appropriate strategies encouraged by the preschool rather than expressing themselves through behaviors such as hitting, biting, screaming, withdrawal from group activities, among others. Importantly, teachers are trained to consider internalizing behaviors, like withdrawal from activities or a child refusing to express how they are feeling as negative strategies in the context of ratings for all three scales. For a different study, involving a subset of the current sample, we also collected daily teacher ratings for the same scales, and these daily ratings were significantly positively correlated with the monthly ratings (*r* = 0.52, p < 0.001), suggesting that these monthly ratings are indexing teachers’ assessments of children’s self-regulatory behavior across that month.

**Coding of Measures of Environmental Stability**

*CPS Involvement*: CPS reports were coded as a sum score of number of reports filed by teachers or school staff while the child was in the program. Just because a report was filed did not mean CPS opened an investigation. However, the fact that teachers and/or home visitors felt an observed event met their criteria for filing a report reflects that staff perceived a potential threat for children in the home warranting CPS involvement, indicative of increased instability within the home.

**Hierarchical Linear Modeling Techniques**

HLM techniques use maximum likelihood estimation to fits a linear function to the observed data while accounting for variation across individuals to estimate the population level rate of change based on the observed data set (Raudenbush & Bryk, 2002; Singer & Willett, 2003). These models are preferable over a repeated measures analysis of variance approach as longitudinal data are inherently nested with time grouped within subject (each individual has a series of outcomes for each time point), and HLM accounts for potential variation in observed outcomes across individuals.

Table S1

*Care placement for children no longer living with their biological parents*

| Type of Care Placement | N (%) |
| --- | --- |
| Foster Care | 22 (69.7) |
| Adoptive Parent | 1 (3.0) |
| Grandparents | 10 (30.3) |

Table S2

| **Monthly Ratings** | **Expresses anger appropriately** | **Copes well with disappointment** |
| --- | --- | --- |
| **Expresses anger appropriately** | - | - |
| **Copes well with disappointment** | 0.74*** | - |
| **Controls impulses** | 0.69*** | 0.71*** |

*Correlations between teacher rating items*

*Note.* ***p < 0.001, **p < 0.01, *p < 0.05, ^†^p < 0.10

Table S3

*Saturated model of teacher ratings of children’s self-regulatory behaviors^1^*

|  | **Model A** | | **Model B** | |
| --- | --- | --- | --- | --- |
| **Fixed Effect** | **β (SE)** | **df** | **β (SE)** | **df** |
| Intercept | 5.24***  (0.17) | 654 | 5.61***  (0.31) | 641 |
| Time | 0.35***  (0.03) | 654 | 0.32***  (0.07) | 641 |
| Living with biological parent(s) | 1.15**  (0.43) | 111 | 1.06*  (0.41) | 102 |
| Number of people in house | 0.07  (0.10) | 111 | 0.03  (0.09) | 102 |
| Number of CPS reports | -0.23  (0.16) | 111 | -0.07  (0.16) | 102 |
| Prior exposure to stress | 0.18*  (0.09) | 111 | 0.06  (0.09) | 102 |
| Family configuration | -0.05  (0.37) | 111 | 0.26  (0.37) | 102 |
| History of abuse | -0.66  (0.41) | 111 | -0.33  (0.39) | 102 |
| Number of moves | -0.11  (0.28) | 111 | 0.01  (0.25) | 102 |
| Number of primary care source changes | -0.02  (0.46) | 111 | -0.48  (0.45) | 102 |
| Living with biological parent(s)*Time | -0.16^†^  (0.09) | 654 | -0.09  (0.09) | 641 |
| Number of people in house*Time | 0.04*  (0.02) | 654 | 0.05*  (0.02) | 641 |
| Number of CPS reports*Time | -0.04  (0.03) | 654 | -0.03  (0.03) | 641 |
| Prior exposure to stress*Time | 0.02  (0.02) | 654 | 0.01  (0.02) | 641 |
| Family configuration*Time | -0.01  (0.08) | 654 | 0.02  (0.09) | 641 |
| History of abuse*Time | 0.01  (0.09) | 654 | -0.04  (0.09) | 641 |
| Number of moves*Time | 0.07  (0.05) | 654 | 0.06  (0.06) | 641 |
| Number of primary care source changes*Time | 0.10  (0.09) | 654 | 0.15  (0.10) | 641 |
| Gender | - | - | 0.91*  (0.39) | 102 |
| Gender*Time | - | - | -0.08  (0.09) | 641 |
| Age | - | - | 0.004  (0.02) | 102 |
| Age*Time | - | - | 0.004  (0.005) | 641 |
| Race – African American/Black | - | - | -0.77^†^  (0.40) | 102 |
| Race – Hispanic | - | - | -0.57  (0.76) | 102 |
| Race - Multi | - | - | 0.40  (0.50) | 102 |
| Race – African American*Time | - | - | -0.16^†^  (0.09) | 641 |
| Race – Hispanic*Time | - | - | -0.06  (0.16) | 641 |
| Race – Multi*Time | - | - | -0.27*  (0.11) | 641 |
| Teacher 1 | - | - | -0.55  (0.42) | 102 |
| Teacher 2 | - | - | 0.45  (0.62) | 102 |
| Teacher 3 | - | - | -1.01*  (0.50) | 102 |
| Teacher 4 | - | - | 1.81  (1.33) | 102 |
| Teacher 5 | - | - | -1.78*  (0.80) | 102 |
| Multiple Teachers Rating | - | - | -0.57  (0.88) | 102 |
| Teacher 1*Time | - | - | 0.07  (0.10) | 641 |
| Teacher 2*Time | - | - | -0.08  (0.14) | 641 |
| Teacher 3*Time | - | - | 0.09  (0.11) | 641 |
| Teacher 4*Time | - | - | 0.02  (0.70) | 641 |
| Teacher 5*Time | - | - | 0.09  (0.13) | 641 |
| Multiple Teachers Rating*Time | - | - | 0.06  (0.20) | 641 |
| **Random Effects** | **Variance** | | | |
| Intercept | 2.79 | | 2.10 | |
| Time | 0.08 | | 0.07 | |
| **Model Fit Statistics** | | | | |
| AIC | 2627.40 | | 2626.38 | |
| BIC | 2729.99 | | 2831.56 | |
| Log Likelihood | -1291.70 | | -1269.19 | |
| r^2^ | 0.27 | | 0.36 | |

*Note.* Saturated models including all predictors and covariates (not including interactions between predictors). Model A: Saturate model including only predictors. Model B: Saturated model including predictors and covariates. The saturated models are not a better fit than the reported model (ps > 0.10). All reported findings remain significant in the saturated models. For analyses including race, Caucasian-White was the comparison group. For analyses including teacher who did the ratings, the teacher who had been a part of the program the longest was the comparison group. ***p < 0.001, **p < 0.01, *p < 0.05, ^†^p < 0.10

Table S4

*Cross-tabulations for covariates with three way interactions*

|  | **Number of household moves** | | | | |
| --- | --- | --- | --- | --- | --- |
|  |  | **0** | **1** | **2** | **3** |
| **Stress** | **0** | 13 | 2 | 2 | 0 |
|  | **1** | 12 | 5 | 0 | 0 |
|  | **2** | 20 | 5 | 1 | 0 |
|  | **3** | 10 | 4 | 2 | 1 |
|  | **4** | 13 | 3 | 0 | 1 |
|  | **5** | 7 | 7 | 1 | 0 |
|  | **6** | 7 | 1 | 1 | 0 |
|  | **7** | 5 | 0 | 0 | 0 |
|  | **8** | 2 | 0 | 0 | 0 |
|  | **9** | 2 | 0 | 0 | 0 |
|  | **10** | 1 | 0 | 0 | 0 |
|  | **Number of changes in care source** | | | | |
|  |  | **0** | **1** | **2** | **3** |
| **CPS Reports** | **0** | 96 | 9 | 2 | 1 |
|  | **1** | 15 | 1 | 0 | 0 |
|  | **2** | 2 | 1 | 0 | 0 |
|  | **3** | 3 | 0 | 0 | 0 |
|  | **4** | 3 | 0 | 0 | 0 |
|  | **5** | 0 | 0 | 0 | 0 |
|  | **6** | 0 | 0 | 0 | 0 |
|  | **7** | 1 | 0 | 0 | 0 |

**References**

Laird, N. M. (1988). Missing data in longitudinal studies. *Statistics in Medicine*, *7*, 305–315.

Raudenbush, S. W., & Bryk, A. S. (2002). *Hierarchical linear models: Applications and data analysis methods*. Sage Publications.

Singer, J. D., & Willett, J. B. (2003). *Applied longitudinal data analysis: Modeling change and event occurrence*. Oxford University Press.
